# Perihematomal Edema Characteristics After Minimally Invasive Surgery in Intracerebral Hemorrhage

**DOI:** 10.1097/CCE.0000000000001344

**Published:** 2025-12-19

**Authors:** Emma D. Frost, Anika Pruthi, Daniel Tonetti, Fred Rincon, Khalid Hanafy, Swarna Rajagopalan

**Affiliations:** 1 Cooper Neurological Institute, Cooper University Hospital, Camden, NJ.; 2 Cooper Medical School of Rowan University, Camden, NJ.; 3 Cooper Department of Neurosurgery, Cooper University Hospital, Camden, NJ.

**Keywords:** intracerebral hemorrhage, minimally invasive surgery, neurocritical care, neuroradiology, perihematomal edema

## Abstract

**OBJECTIVES:**

Perihematomal edema (PHE) impacts recovery after spontaneous intracerebral hemorrhage (sICH). How minimally invasive surgery (MIS) affects PHE compared with medical management and conventional surgical management (craniotomy or decompressive craniectomy), and whether this relates to functional outcomes remains poorly understood.

**DESIGN:**

In this single-center observational study including 40 patients (MIS *n* = 16, medical management *n* = 13, conventional surgical evacuation, *n* = 11), we assessed PHE volumes and functional outcomes after MIS for sICH and compared them with medical management and conventional surgical management. We collected data retrospectively, calculating hematoma and perihematomal volumes using the validated ABC/2 method (A = maximal diameter, B = orthogonal diameter, C = slice count × thickness). We used linear mixed modeling in IBM SPSS (statistical software package) to detect differences in peak PHE, interaction between PHE and days, and differences in functional outcomes across the three treatment groups. ICH score was a covariate in all modeling. The outcomes were peak PHE volume, PHE trajectory comparison across treatment groups, and 90-day functional outcome. Research was institutional review board approved and conducted in accordance with the ethical standards of the responsible committee on human experimentation (institutional or regional) and with the Helsinki Declaration of 1975.

**SETTING:**

Study was conducted in a single tertiary care center with 24-hour neurocritical care and neurosurgical services.

**INTERVENTIONS:**

Patients were grouped based on which intervention they underwent. As study was conducted retrospectively, intervention (medical management, surgical evacuation, MIS) were determined based on clinical appropriateness.

**MEASUREMENTS AND MAIN RESULTS:**

We collected data retrospectively, calculating hematoma and perihematomal volumes using the validated ABC/2 method. PHE trajectory was compared with 90-day functional outcome and time across all groups. MIS was associated with *significantly lower* peak PHE burden, compared with medical and conventional surgical treatment groups, after accounting for ICH score (*F* [2, 118] = 7.26; *p* = 0.001). PHE evolved over time, across all treatment groups (*F* [9, 118] = 2.26; *p* = 0.023). MIS tended to peak earlier, but the shape of the PHE trajectory over time did not differ significantly between groups (*F* [16, 118] = 1.18; *p* = 0.295). MIS was associated with better functional outcomes (90-d modified Rankin Scale [mRS]) based on treatment type (*p* < 0.001) with the MIS group having the lowest average mRS 2.3 ± 1.49, medical management group having an average of 3 ± 2, and the standard evacuation group having average of 4.3 ± 1.4, after accounting for ICH score. Higher baseline ICH score also independently associated with worse outcome (*F* [1, 143] = 4.37; *p* = 0.038). While the sample size was small and results are exploratory, together the findings suggest that treatment modality for sICH influences both long-term functional outcomes and PHE burden, independent of baseline ICH severity. These findings suggest the temporal profile of edema resolution, rather than merely its volume, may be a key mechanism underlying MIS benefits in sICH management.

**CONCLUSIONS:**

In this observational exploratory study, MIS, compared with both medical therapy and conventional surgery, was associated with reduced peak of PHE and better 90-day functional outcome, independent of baseline sICH severity. The difference in temporal trajectory of edema, while may be clinically meaningful, was not statistically significant between treatment strategies. Larger prospective studies with standardized imaging protocols are needed to validate these observations and explore their implications for optimizing post-ICH care.

**KEY POINTS**

**Question**: How does perihematomal edema (PHE) evolve after minimally invasive surgery (MIS) for spontaneous intracerebral hemorrhage (sICH), and how does it compare with medical and conventional surgical management in relation to functional outcomes?

**Findings**: In this single-center observational study of 40 patients, MIS was associated with a significantly lower peak PHE burden and better 90-day functional outcomes compared with medical therapy and conventional surgery, independent of baseline ICH score. While MIS tended to produce earlier edema peaking, temporal trajectories of PHE evolution were not statistically different across groups.

**Meaning**: MIS may mitigate secondary brain injury by reducing the overall magnitude of PHE and improving functional outcomes in sICH, even in patients with moderate hematoma volumes. Larger, prospective studies with standardized imaging are needed to clarify the temporal dynamics of PHE resolution and to validate these exploratory findings.

Spontaneous intracerebral hemorrhage (sICH) remains a devastating neurologic emergency with high morbidity and mortality despite medical advances (1). The acute brain injury following sICH comprises both primary brain injury from the initial hemorrhage and secondary brain injury (SBI) arising from subsequent inflammatory cascades, oxidative stress, and local mass effect (2).

Perihematomal edema (PHE) has emerged as a critical component of SBI following sICH. Its formation involves complex, multifactorial, mechanisms such as hydrostatic pressure shifts, osmotic gradients, tissue ischemia, hemolysis, and cytotoxic edema from neuronal death and iron deposition (3–5). While PHE development has been extensively studied in conservatively managed patients, its dynamics following surgical intervention remain poorly characterized (6).

Currently, there are limited treatment for reducing SBI after sICH, with interventions typically aimed at managing blood pressure, mitigation of hyponatremia, osmotherapy, correction of coagulopathies, and glucose control. Minimally invasive surgery (MIS) has shown promise in improving functional outcomes after sICH compared with standard medical management or conventional surgery and craniotomy or decompressive craniectomy. The Trial of Early Minimally Invasive Removal of Intracerebral Hemorrhage (ENRICH), which enrolled mostly cortical hemorrhages, demonstrated that early MIS intervention within 24 hours of symptom onset significantly improves functional outcomes at 180 days (7). However, the underlying mechanisms driving this improvement remain largely unexplored, particularly regarding how MIS affects PHE development and trajectory. Traditional craniotomy for hematoma evacuation often leads to surgical trauma, which may exacerbate inflammation and edema (8). In contrast, MIS techniques offer reduced tissue disruption while still achieving effective clot removal. These differences could fundamentally alter PHE evolution.

We hypothesize that MIS might lead to reduced and earlier peaking of PHE, defined as the largest measured PHE volume, compared with standard approaches, and that this modified edema trajectory correlates with improved functional outcomes. Establishing the precise temporal evolution of PHE after MIS is critical for optimizing the type and timing of adjunctive interventions, such as osmotic, anti-inflammatory, or additional surgical treatments, and may clarify mechanisms by which these treatments influence recovery.

This study investigates the early trajectory of PHE after MIS evacuation of sICH, compared with standard medical and conventional surgical management. We hypothesized that MIS reduces PHE after sICH, and that it correlates with better short-term functional outcomes, measured by modified Rankin Scale (mRS) at 90 days.

## METHODS

### Patient Selection

We created a registry of consecutively admitted adult patients (≥ 18 yr old) with a diagnosis of supratentorial sICH who underwent minimally invasive hematoma evacuation. These patients were then matched with individuals from the institution’s retrospective database of all sICH patients treated between 2016 and the present, based on ICH score, which accounted for age, volume of hematoma, Glasgow Coma Scale score, intraventricular extension, and location.

This approach allowed for comparison between patients who underwent MIS, those who received medical management, including management targeted at control of hypertension, hyperosmolar therapy for symptomatic edema, and those who underwent nonminimally invasive surgical evacuation.

### Inclusion Criteria

All patients with a sICH who underwent MIS hematoma evacuation during their hospitalization were included in this study. All MIS patients had evacuation by the same neurosurgeon using stereotactic image guided evacuation. The inclusion criteria for MIS at our institution are a clinically disabling deficit, determined by both the treating neurointensivist and neurosurgeon, which differed from the ENRICH trial, which only enrolled patients with hematoma volumes (H_v_) exceeding 30 mL. The surgical technique for evacuation was up to the treating attending neurosurgeon’s preference. This study is an observational, retrospective review in which data were collected in a HIPAA-compliant manner. The data includes all patients who fit the inclusion criteria from September 2022 to June 2024. Matched controls were then obtained for those patients based on ICH score. Patient demographics are shown in **Table 1**.

**TABLE 1.** Baseline Characteristics of Patients With Intracerebral Hemorrhage by Treatment Approach

Characteristic	Minimally Invasive Surgery Group (*n* = 16)	Medical Management Group (*n* = 13)	Conventional Surgery (*n* = 11)
Demographics			
Age, yr, median (IQR)	61 (26–74)	59 (43–74)	60 (35–77)
Medical history, *n* (%)			
Hypertension	12 (75)	9 (69)	7 (64)
Active smoking	5 (31)	2 (15)	3 (27)
Anticoagulation use	1 (6)	2 (15)	4 (36)
Antiplatelet use	6 (38)	7 (54)	1 (9)
ICH characteristics			
Location, *n* (%)			
Cortical	11 (69)	6 (46)	4 (36)
Deep	3 (19)	5 (38)	3 (27)
Both	2 (12)	2 (15)	4 (36)
ICH score, median (IQR)	1 (0–3)	1 (0–2)	2 (1–3)
Preoperative ICH volume, mL, mean ± sd	21.58 ± 13.01	14.38 ± 7.96	33.46 ± 14.44
Preoperative perihematomal edema volume, mL, mean ± sd	11.16 ± 8.42	8.16 ± 6.16	15.93 ± 10.89
Time from diagnosis to surgical intervention, hr ^a^	11:44		9:48

ICH = intracerebral hemorrhage, IQR = interquartile range.

a Time from diagnosis to surgical intervention reported for surgical cohorts only; not applicable to medical management.

### Exclusion Criteria

Those with a history of prior ICH, ICH following a procedure or trauma, ICH secondary to a tumor or known arteriovenous malformation rupture, and those with additional bleeds such as ICH, subdural, epidural, or subarachnoid were excluded from the study. Patients with a prior history of large ischemic stroke, traumatic brain injury in the past were excluded. This exclusion was due to the potential for the inflammatory cascade associated with these underlying causes to alter the natural progression of PHE after ICH.

### Data Collection

All patients who underwent MIS hematoma evacuation at Cooper University Hospital were included in the study unless ruled out based on exclusion criteria listed above. Data elements included patient demographic data (age, sex, race), clinical data (pre-stroke mRS, National Institutes of Health Stroke Scale), imaging findings, surgical dates, and times were abstracted into a web-based, HIPAA-compliant data management platform. All imaging findings were initially read by a board-certified neuroradiologist. sICH volume and PHE volume were calculated by the A × B × C/2 method (A = maximal diameter, B = orthogonal diameter, C = slice count × thickness) by a single trained rater using a standardized protocol. Researchers were not blinded to whether a patient had undergone MIS or what their mRS at discharge was. Digital volumetric analysis is not used due to software constraints and inconsistent blood/edema separation, as determined by three independent research team members. CTs included the presenting scan and postoperative days 0–10 as available; intervals were clinically determined and therefore not standardized and not all subjects had data points for each of those days. The primary outcome measures were PHE volume, PHE trajectory across days, and mRS at 90 days, which were segmented on a nominal scale of 0–6 with 0 being the best outcome of no function deficit and 6 being the worst, death.

### Statistical Analysis

The peak PHE day per patient was defined as the postoperative day with the maximum PHE volume. Patients were stratified in three treatment groups, MIS evacuation, medical management, and conventional surgery. We used SPSS Mixed Models (v29.0.2.0; IBM Corp, Armonk, NY) to fit two linear mixed-effects models:

1) PHE peak and trajectory model: The dependent variable was daily PHE peak volume. Fixed effects included treatment group, day, and the group and day interaction, with baseline ICH score entered as a covariate. Patient identification (ID)was specified as the subject variable, with day treated as the repeated measure with autoregressive 1 (AR1) covariance. Baseline ICH score was included as a covariate. An AR1 covariance structure was specified to account for within-subject correlation across repeated days.
2) Functional outcome model: The dependent variable was mRS at 90 days. Fixed effects included the treatment group, and baseline ICH score was included as a covariate. A random intercept was included for the subject, and variance components were used as the covariance structure.

### Ethics

This study was approved by the local institutional review board (IRB), with a waiver of informed consent, as part of the standing registry for the collection and use of retrospective data regarding all ischemic and hemorrhagic strokes, including ICH, which was last addended on January 20, 2025 (IRB No. 20-371). All research was completed in accordance with the ethical standards of the responsible committee on human experimentation (institutional or regional) and with the Helsinki Declaration of 1975.

### Image Analyses

H_v_, total perihematoma volume, and perihematoma edema volume were measured in mL.

## RESULTS

### PHE Peak and Trajectory Between Groups

**Figure 1.**
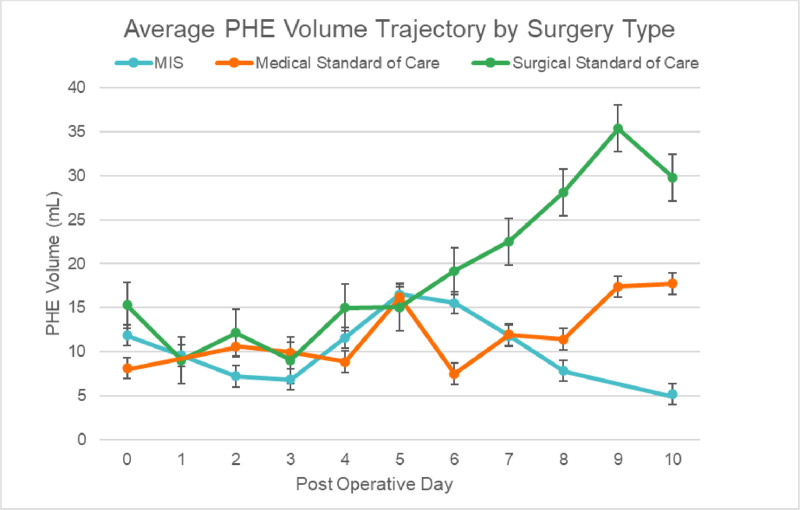
Mean perihematomal edema (PHE) volume by day. Day 0 = day of bleed (medical) or day of surgery (minimally invasive surgery [MIS]/conventional surgery). *Error bars* = sem.

In the PHE peak and trajectory model, treatment groups differed significantly in overall PHE burden (*F* [2, 118] = 7.26; *p* = 0.001) and day-to-day PHE variation (*F*[9, 118] = 2.26; *p* = 0.023). MIS patients had significantly lower overall PHE burden compared to medical management or conventional surgery. MIS patients also reached peak PHE earlier (2.25 ± 2.62 d) than medical (3.31 ± 3.17) and conventional surgery (2.90 ± 3.11) groups. The group and day interaction was not significant (*F*[16, 118] = 1.18; *p* = 0.295), suggesting that temporal trajectories of PHE did not differ significantly between groups, despite the earlier peaking in MIS. Baseline ICH score did not predict PHE peak burden or timing (*F*[1, 118] = 1.04; *p* = 0.310; **Table 2**). The medical management group showed a distinct biphasic PHE peak pattern, with peaks around days 5 and 10—a pattern not observed in either the MIS or conventional surgery cohorts, visualized in **Figure 1**.

**TABLE 2.** Type III Tests of Fixed Effects^a^ Regarding Peak Perihematomal Edema

Source	Numerator *df*	Denominator *df*	*F*	Significant
Intercept	1	118	37.751	< 0.001
Group	2	118	7.262	0.001
Day	9	118	2.262	0.023
Group × day	16	118	1.178	0.295
Intracerebral hemorrhage score	1	118	1.041	0.31

*df* = degrees of freedom.

a Dependent variable: perihematomal edema peak volume.

### Relationship Between PHE Timing and 90-Day Functional Outcome

In the functional outcome model, treatment modality was a significant predictor of 90-day functional outcome, with clear differences across groups (*F*[2, 143] = 16.81; *p* < 0.001). The MIS group achieved the best functional outcomes (mean mRS 2.3 ± 1.49), followed by the medical management group (mean mRS 3 ± 2), while the conventional surgical group had the worst outcomes (4.3 ± 1.4). Baseline ICH score was also independently associated with worse functional outcome (*F*[1, 143] = 4.37; *p* = 0.038; **Table 3**), consistent with previously validated findings (9, 10).

**TABLE 3.** Type III Tests of Fixed Effects^a^ Regarding Modified Rankin Scale at 90 Days

Source	Numerator *df*	Denominator *df*	*F*	Significant
Intercept	1	143	166.954	< 0.001
Group	2	143	16.812	< 0.001
Intracerebral hemorrhage score	1	143	4.371	0.038

*df* = degrees of freedom.

a Dependent variable: 90-d modified Rankin Scale.

Individual data can be found in **Appendix 1** (https://links.lww.com/CCX/B585).

While the sample size was small and results are exploratory, together the findings suggest that treatment modality influences both long-term functional outcomes and PHE burden, independent of baseline ICH severity.

## DISCUSSION

In this study, we compared patients with sICH who underwent MIS evacuation to those who received medical management or conventional surgery. Our initial hypothesis was that MIS would both reduce the magnitude of PHE and accelerate its peaking, with earlier peaks correlating with improved functional outcomes. Our results provide partial support for this hypothesis. We found that MIS patients demonstrated lower overall burden of PHE compared with the other two treatment groups. This reduction in edema burden was the most robust and statistically significant finding in our analysis. Notably, baseline ICH score did not predict PHE burden or peak timing in our cohort, reinforcing that the observed edema differences were driven by treatment modality rather than initial hemorrhage severity. The group and day interaction, that is, the temporal trajectory of PHE was not statistically significant, suggesting that while MIS lowers cumulative edema volume, it may not fundamentally alter the temporal dynamics of edema evolution. Although this difference did not reach significance, the MIS patients tended to peak in edema earlier (Fig. 1), and it may be clinically meaningful. An earlier resolution of edema could reduce the duration of mass effect and local tissue distortion, potentially allowing earlier recovery of perihematomal brain function. This observation is biologically consistent with the concept that MIS evacuates clot and reduces the substrate for secondary injury—limiting erythrocyte lysis products such as hemoglobin and iron—while minimizing collateral parenchymal disruption due to its smaller operative footprint. The result is a lower peak edema burden without directly altering the inflammatory and vascular cascades that drive edema evolution. We did not find that MIS altered the temporal course of edema; however, it is possible that our study was underpowered to detect more subtle trajectory differences, particularly given the variability of edema volumes and wide CIs at later time points.

The pathophysiology of PHE remains incompletely understood. Our study supports emerging data suggesting PHE evolves in two phases—an early phase (protein/ionic shifts) and a late phase (erythrocyte lysis/iron-mediated toxicity) over ~21 days (1) (Fig. 1). In our cohort, we also describe PHE trajectories in MIS and conventional surgery groups—early-phase PHE in the MIS group (potentially adaptive), and a prolonged course of PHE in the conventional surgery group, however, our follow-up was too short to confirm late-phase peaks or to compare time-to-maximum PHE across groups, and this remains to be explored. These dynamics are intriguing and may caution against blanket early suppression of PHE: aggressive osmotherapy or steroids in the first 48 hours may blunt potentially adaptive early neuroinflammation and could partly explain negative trials focused solely on reducing PHE (11). A distinctive finding in our study was the biphasic PHE pattern in the medical management group, with peaks around days 5 and 10, a pattern not seen in either MIS or conventional surgery. This may reflect ongoing secondary injury processes in the absence of surgical clot evacuation and warrants further study.

Perhaps most importantly in our exploratory study, MIS was associated with improved 90-day functional outcomes compared with both medical and conventional surgical management, independent of baseline ICH score. This association was observed even in patients with relatively modest H_v_ (mean ~22 mL) when accompanied by clinically disabling deficits. These findings suggest that the potential mechanisms by which MIS improves functional outcomes may extend beyond radiographic edema reduction and include alleviation of mass effect, improved intracranial pressure control, reduced neurotoxicity from blood breakdown products, restoration of local microcirculation, overall reduced secondary inflammatory processes, and enhanced potential for neuroplastic recovery. Our study was not designed to directly assess these hypotheses, and we cannot draw definitive conclusions regarding these mechanisms. The fact that baseline ICH score also independently predicted outcome (*p* = 0.038;Table 3) reinforces its established role as a clinical severity measure and validates the internal consistency of our model. Our findings, although exploratory due to small sample size and retrospective nature of the study, should prompt further investigation into a large sample size.

Overall, MIS appears to reduce the overall burden of SBI while allowing expected inflammatory/vascular cascades to proceed. The primary benefit is a lower peak edema volume, not a demonstrable change in trajectory, though the trend toward earlier peaking warrants study. Selection should consider clinical deficit in addition to hematoma size; even smaller hemorrhages may benefit from MIS when deficits are disabling. Retrospective multicenter studies with dense, standardized imaging through ~21 days are needed to assess time-to-peak explicitly and delineate late-phase PHE.

This single-center cohort (*n* = 40) is exploratory with limited generalizability. Groups were imbalanced: the medical cohort had lower mean ICH scores (higher-severity patients proceeded to surgery), whereas the conventional surgical cohort had higher ICH scores; hematoma location also differed across groups. Although we matched for and statistically adjusted for ICH score, residual confounding is possible.

Automated volumetric tools failed to accurately differentiate blood from edema, necessitating manual ABC/2 measurements, which, while validated, introduce potential variability, and because treatment group assignment was evident on CT scans, measurements could not be performed in a blinded manner. The observational design further limited our ability to standardize imaging intervals, clinical management protocols, or timing of interventions and scans may have missed true peak windows. Most MIS procedures occurred within 24 hours (mean, 13.79 hr) but transfers obscured ictus-to-surgery interval. Our institution does not apply strict volumetric or clinical cutoffs for MIS selection, limiting reproducibility and generalizability to other centers. Due to a small sample size, propensity score matching was not feasible; we sampled controls to mirror MIS ICH score distribution and adjusted for ICH score, but residual bias cannot be excluded.

Despite these limitations, our study provides valuable preliminary evidence that MIS reduces PHE burden and is associated with better 90-day functional outcomes independent of baseline sICH severity, compared with medical and surgical standard of care. These data highlight the need for larger, prospective, and multi-institutional studies with standardized frequent imaging and treatment protocols to clarify the mechanistic relationship between edema dynamics including magnitude, peak, resolution of PHE, and functional recovery after sICH.

## CONCLUSIONS

In this single-center observational exploratory study, MIS, compared with both medical therapy and conventional surgery, was associated with reduced peak of PHE and better 90-day functional outcomes, independent of baseline sICH severity. The difference in the temporal trajectory of edema, while it may be clinically meaningful, was not statistically significant between treatment strategies. Larger prospective studies with standardized imaging protocols for a longer duration of time are needed to validate these observations and explore their implications for optimizing post-ICH care.

## Supplementary Material
