# Supplementary material for: Perihematomal Edema Characteristics After Minimally Invasive Surgery in Intracerebral Hemorrhage
Source: Crit Care Explor. 2025 Dec 19;7(12):e1344. doi: 10.1097/CCE.0000000000001344 (PMC12721792; doi:10.1097/CCE.0000000000001344)
Supplement: Supplementary file 1 [file cc9-7-e1344-s001.pdf]

Dummy-ID	MIS vs MM vs SM	ICH Score	Age (at time of procedure)	ICH Volume at adm (mL)	Date Surgery	PHE on pre-evacuation mL
1	1	1	61	16.2	2/25/24	1.896
2	1	2	70	9.1	4/4/24	2.73
3	1	0	74	15.3	04/19/24	1.33
4	1	3	58	26.99	09/16/22	26.29
5	1	1	41	11.42	11/17/22	16.59
6	1	3	69	4.85	01/09/23	7.086
7	1	1	66	48.2	1/21/23	10.35
8	1	2	39	13.46	3/27/23	7.72
9	1	1	62	21.4	1/25/23	16.8
10	1	2	66	50	6/30/23	33.7
11	1	2	63	33.6	7/28/23	6.1
13	1	1	45	22.2	12/17/23	7.1
14	1	1	53	34.8	01/04/24	15.5
15	1	1	60	8.53	6/21/24	5.75
16	1	3	56	14.72	6/15/24	16.78
17	1	1	54	24.4	1/4/25	14.4
		1				
Medical Management group						
18	0	1	62	15.4		1.9
19	0	1	71	5.2		0.5
20	0	2	45	10.3		4.3
21	0	2	70	10.2		5.3
22	0	2	74	17.5		12.3
23	0	2	56	25.6		6.7
24	0	0	58	13.5		4.1
49	0	1	47	2.4		3.5
26	0	1	60	11.8		19.9
27	0	1	43	26.8		18.7
28	0	2	55	26.9		9.1
44	0	1	61	13.4		6.7
45	0	1	67	7.9		13.1
Totals		1	59.15384615	14.37692308		8.161538462
				7.958030938		6.155964379
Surgical standard of care						
29	2	1	66	45	10/18/17	15.2
30	2	1	68	50.2	5/15/20	18.7
31	2	3	55	36.7	5/23/17	21.8
32	2	2	35	45.8	4/27/22	7.3
33	2	2	61	22.1	11/26/22	3.1
25	2	1	67	33.7	3/16/18	3.6
34	2	3	68	12.8	5/24/22	12.1
35	2	3	61	50.8	5/18/25	37.3
36	2	2	77	39.1	11/19/24	31.5
37	2	1	67	14.2	2/16/25	14.9

	38	2	2	37	17.7	5/11/23	9.7
Totals			2	60.18181818	33.46363636		15.92727273
					14.43580775		10.89688863
				possible DHC M	21643331	120cc	
				nonminimally in	10832156		
				MIS	10889067		
					10939015		
	46	0	1	64	2.4		1.5

PHE volume POD0	PHE volume POD 1/ PBD 1	PHE volume POD 2/ PBD2	PHE volume POD3/PBD 3	PHE volume POD4 /PBD4	PHE vol POD5 /PBD5	PHE volume POD6 / PBD6
10.407	8.41					10.946
9.6		3.19		9.63	3.98	
8.99	4.38					
8.495		1.762	1.68			
0	2.08					
13.155	3.136	11.82		6.403		
12.1	18.1			27.5		
11.87	8.715	9.145	16.08	11.953	12.34	
0	3.4	6.4	13.9	25.9	28.7	
24.7	16			17.4		
20.4						14.8
9.4	8.5	10.2	19.4	17.1	17.3	19.7
9.4						
7.736	5.001					
2.2747	1.975	5.599	7.087			1.928
5						

	11.6	17.9	19		19.8	19.2
	0.3	1.5	2.2	2.4	3.4	4.7
	8.9					
	7.5		7.1			
	13.2					
	12.6		11.7			
	3.9	4.5	4.6		4.7	
		4.9			2.2	
	16	14.5				
	25.6			30		
	6.9					
	7		8.7			
	14.1	16.4				

	2.4	0	0	0		
10.1		47	18.3		11.9	
16.4	45.6					53.5
0	0	1.5				2.9
0	1		14.9			9.3
14.5	16.9		11.4			
5.1		6.3				
5.7	11.2		33.5	22.5	26.3	24.4
11.5		24.8		22.7	24	
21.8	14	14.46	19		14.6	

1.8 8

1.7 0.7 0.9

PHE volume POD7/ PBD7	PHE Volume POD 8/PBD8	PHE Volume POD 9/ PBD9	Recommended Discharge location	MRS at discharge	MRS at 90 days	Hx HTN
		5.656	4	4	4	0
			0	2	2	1
			2	3	1	1
1.22			0	4	1	1
			1	3	2	1
6.243			1	4	0	1
			1	3	3	0
16.075			1	3	1	0
			1	3	1	1
			2	3	3	1
			3	4	2	1
			4	6	6	1
			1	3	2	1
			1	3	3	1
		4.729	3	5	4	1
			1	3	2	1
23.3	22.2	24.9	4	6	6	0
4.8	6		4	6	6	1
		5.2		3	3	1
6.2			4	6	6	1
	24	23.2	3	5	5	1
				4	1	0
				2	2	1
			1	2	2	1
			1	3	3	1
			2	3	3	0
			2	3	1	1
			0	0	0	1
			1	2	1	0
						9
			3	5	5	1
		29.8	3	5	6	1
47.4	47.8		2	4	4	1
			2	3	3	0
			2	4	4	0
11.9			2	4	2	1
			5	5	6	1
25.6	22.9		2	5	4	1
			2	4	4	1
27.6			4	6	6	0

	2	5	3	0<br>7

Home 0
Acute rehab 1
SAR 2
LTAC 3
Death 4
Hospice 5

2.1	1	2	0	

Hx Smoking	Hx Afib	Hx AUD	On AC?	On Antiplatele	Antiplatelet ag	Location of ble
2	0	0	0	0		1
0	0	0	0	1	asprin	1
0	0	0	0	0		1
1	0	0	0	0		1
0	0	1	0	0		1
0	0	0	0	0		1
2	1	0	1	0	Warfarin	1
0	0	0	1	1	asprin/plavix	2
0	0	0	0	1	asprin/brillinta	1
0	0	0	0	1	asprin/plavix	2
1	0	0	0	1	asprin/plavix	1
0	0	0	0	0		0
1	0	0	0	0	no	1
0	0	0	0	1	asprin	1
1	0	0	0	0		0
1	0	0	0	0		1
0	0	1	0	1	asprin	0
0	1	0	1	0		0
1	0	0	0	1	aspriin	0
0	0	0	0	1	plavix	0
0	0	0	0	1	aspirin	2
0	0	1	0	0		2
0	0	0	0	0		1
0	0	0	0	1	aspirin	0
0	0	0	0	0		1
0	0	0	0	0		1
0	0	0	0	1	aspirin	1
1	0	0	0	1	ASA + Plavix	1
0	0	0	0	0		1
2= active	1	2	2	7		5 deep
0= former						6 cortical
11= never						2 both
2	0	0	0	0		1
0	1	0	1	0		2
0	0	0	0	0		2
2	0	0	0	0		1
0	0	0	0	0		0
0	0	0	0	0	0	2
1	0	0	0	0		2
1	0	0	1	0		0
0	1	1	1	1	aspirin	1
0	0	0	1	0		1

	1	0	1	0	0		0
3 current		2	2	4	1		
2 former						3 deep	
6 never						4 cortical	
						4 both	

Deep =0
Cortical =1
Both = 2

eed
